# Design, Methods, and Participation in Riksmaten Young Children—A Swedish National Dietary Survey

**DOI:** 10.1016/j.cdnut.2024.102150

**Published:** 2024-04-30

**Authors:** Helena Bjermo, Emma Patterson, Jessica Petrelius Sipinen, Sanna Lignell, Karin Stenberg, Elin Larsson, Anna Karin Lindroos, Jakob Ottoson, Eva Warensjö Lemming, Lotta Moraeus

**Affiliations:** 1 Department of Risk Benefit Assessment, Swedish Food Agency, Uppsala, Sweden; 2 Department of Global Public Health, Karolinska Institutet, Stockholm, Sweden; 3 Department of Internal Medicine and Clinical Nutrition, The Sahlgrenska Academy, University of Gothenburg, Gothenburg, Sweden; 4 Department of Food Studies, Nutrition and Dietetics, Uppsala University, Uppsala, Sweden; 5 Medical Epidemiology, Department of Surgical Sciences, Uppsala University, Uppsala, Sweden

**Keywords:** children, dietary assessment, national dietary survey, web-based, recruitment, biomonitoring

## Abstract

**Background:**

National dietary surveys provide essential data for risk benefit assessments of foods and nutrients, for management and policy development. Physical activity measurement and biomonitoring can provide important complementary data but are less commonly included.

**Objectives:**

This study aimed to describe the study design and methods of the cross-sectional Swedish national dietary survey Riksmaten Young Children (Riksmaten småbarn), of children aged 9 mo, 18 mo, and 4 y. Participation/dropout rates for the 2 older age groups are also presented. The impact of different recruitment strategies is discussed.

**Methods:**

Children (*N* = 16,655) were randomly selected from the population register; invitations to guardians were sent by post and where possible, followed up by telephone. Food intake was assessed by a 2-d food diary and/or questionnaire. Height and weight were reported after measurement. Physical activity (accelerometery, 7 d) and stool, blood, and urine samples were assessed in subgroups.

**Results:**

Food consumption data were collected in 1828 children (11% of the invited; 18 mo: *n* = 1078, and 4 y: *n* = 750). Of participants also in subgroups, 71% provided physical activity data (*n* = 1307), 60% stool samples (*n* = 630), and 51% blood and/or urine samples (*n* = 593). The study population represented all geographic regions and types of municipalities in Sweden, but participating households had both higher education level and higher income than the target population. Only minor differences were seen in participation rates between recruitment via post and telephone compared with those through post only (12% compared with 10%). Repeated contact attempts were needed for the majority of participants (65%). Despite the low-participation rate, 99% of the participants completed the study once started.

**Conclusions:**

Although it was a challenge to recruit participants, Riksmaten Young Children provides a substantial amount of information at national level, representative in terms of sex, geography, and family structure. The underrepresentation of households with lower socioeconomic position must be considered when generalizing results.

## Introduction

National dietary surveys are an essential source of data on food consumption and nutritional status in population groups. The data from dietary surveys are used in many ways, for example, when conducting risk and/or benefit assessments of foods, nutrients and unwanted substances, or when forming public health policy and food regulations. The latest national dietary survey conducted by the Swedish Food Agency included adolescents aged 12–18 y. That survey found that one-third of the girls between 14 and 18 y were at risk of iron deficiency [1] and that ∼40% of energy came from discretionary foods and foods high in added sugar and saturated fat [2]. These insights are important when prioritizing public health interventions and deciding which measures should be taken. Data from European countries are also shared with the European Food Safety Authority (EFSA) to be used in risk analyses, which underpin legislation and policymaking in Europe [3]. Such risk analyses can, for example, involve food additives, pesticides, dietary supplements, and fortified foods.

Preschool children are a sensitive group when it comes to establishing food habits and preferences [4,5], and nutrient needs are high [6]. Moreover, because Swedish school children are exposed to free school meals, there is a possibility to mitigate diet-related social inequalities in this population [7]. Children are also especially susceptible to the adverse effects of harmful substances owing to their rapid growth and development. However, nationally representative detailed information on dietary habits in children aged <4 y in Europe is rare, and Sweden lacks this information entirely [8]. There is also a need for updated information on children aged 4-y, which in Sweden were last surveyed in 2003 [9]. Therefore, the Swedish national dietary survey of young children (Riksmaten Young Children, “Riksmaten småbarn”) was designed to collect data on children of ages 9 mo, 18 mo, and 4 y.

National dietary surveys conducted by the Swedish Food Agency provide detailed information on nutrient and energy intake and food choices. In addition, physical activity assessment, and biomarkers from blood and urine samples are included. The latter was in order to capture nutritional status with respect to vitamin D, folate, iron, and iodine and total exposure to contaminants such as polychlorinated biphenyls, perfluoroalkyl and polyfluoroalkyl substances, lead, and mercury [2,1,[10], [11], [12]]. In Riksmaten Young Children, collection of stool samples was added to the method, in order to investigate antibiotic resistant bacteria, resistance genes, and enteric viruses.

### Aim

The aims of this study were to describe the study design and methods of the Swedish national dietary survey Riksmaten Young Children; to present an analysis of participation/dropout rates for the children aged 18 mo and 4 y; and to discuss the impact of different recruitment strategies.

## Methods

### Study design

Riksmaten Young Children is a national cross-sectional survey of young children aged 9 mo, 18 mo, and 4 y. Because the data collection in the 9-mo-olds is still ongoing, with the assessment being less detailed than that in the older groups, they are described only briefly, and the results are presented based on children aged 18 mo and 4 y.

Data from each age group were collected sequentially (1 age group per year), starting with the eldest children, between early autumn and late spring, starting in 2021. For simplicity, we will hereafter refer just to “participants,” but, obviously, in most cases, information about the children was provided by parents/guardians. The following data were collected from all 18-mo-old and 4-y-old participants: food consumption via a web-based food diary for 1 weekend and 1 weekday; food habits via a web-based questionnaire; anthropometric data reported via guardians after a routine visit to a Children’s Healthcare Centre; and objectively measured physical activity. Blood, urine, and stool samples were collected in subgroups. For children aged 9 mo, food intake was assessed by a food frequency questionnaire only, and stool samples were collected in a subgroup. A food diary was deemed too burdensome as many 9-mo-old children are still partly breastfed and their food intake was not so extensive. The sample selection, study design, and recruitment strategy were broadly similar for all 3 age groups. Ethical approval was obtained from the Swedish Ethical Review Authority (2020-05293).

### Pilot survey

In preparation for the main survey, a pilot survey was conducted in the target population. All 3 age groups (9 mo, 18 mo, and 4 y) were surveyed in parallel between March and May 2021. The sample was ordered from the Swedish state personal address register and included 1000 children from each age group. The recruitment process and survey methods were tested, and apart from some adjustments, the same methods were used in the main survey. The adjustments made were as follows: *1*) apart from the food diary and questionnaires, all parts of the survey were made voluntary, providing more flexibility; *2*) the reimbursement rate for those who participated in blood sampling was increased; *3*) written simple individualized feedback based on the food diary and physical activity measurements was offered; and *4*) the sample size was increased as a result of the lower than expected participation rates. These changes were approved by the Swedish Ethical Review Authority (2021-03591).

### Data collection

#### Population

**FIGURE 1:**
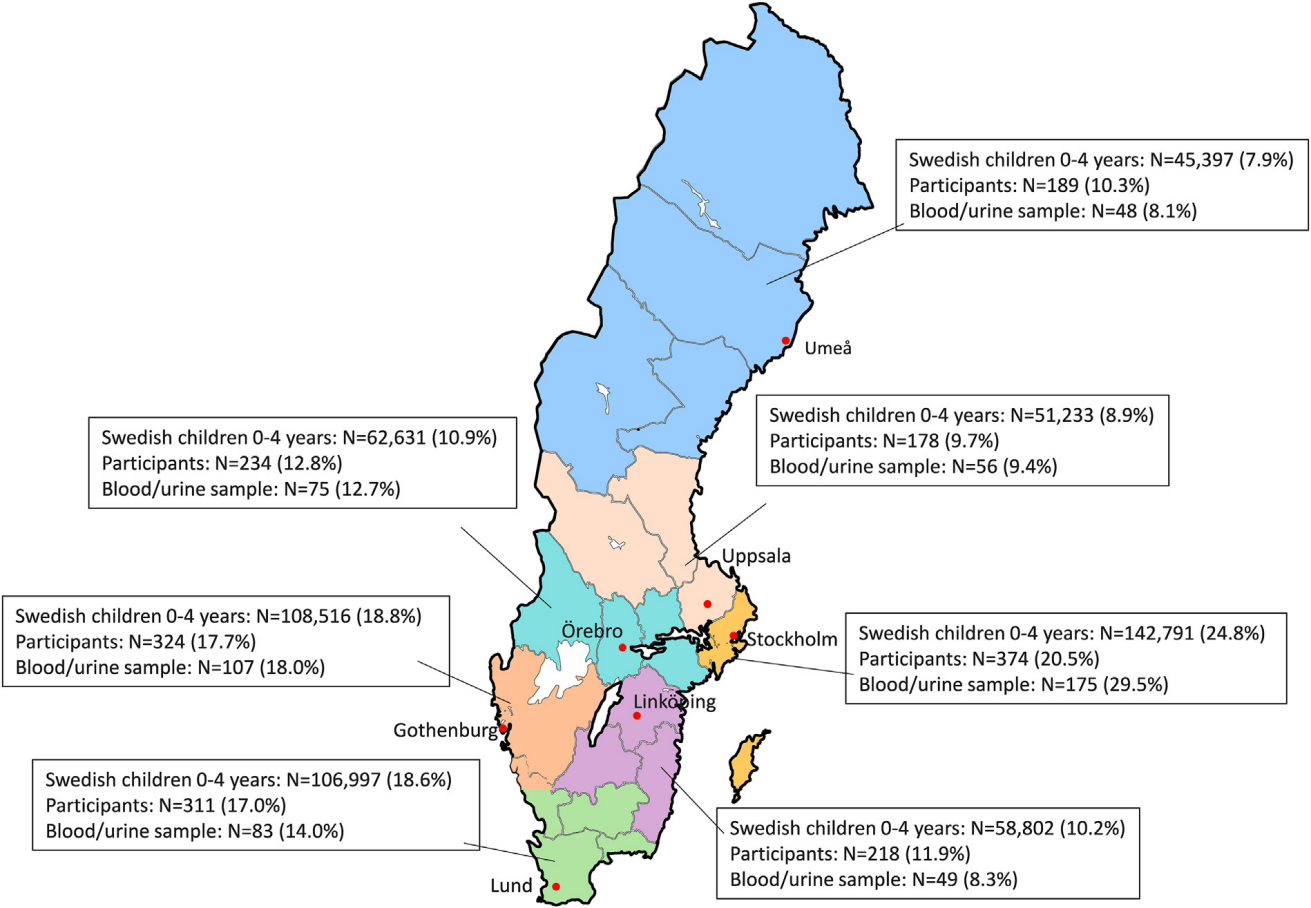
Geographic distribution of the participants in Riksmaten Young Children (*n* = 1828), participants donating blood and/or urine (*n* = 593), and the Swedish population of children aged 0–4 y (*N* = 576,367). The Swedish population is based on data from Statistics Sweden from 2022 [13].

For the main survey, register-based random samples were drawn by the government agency Statistics Sweden. The samples were stratified based on the level of education achieved and at household level. The goal was to include ∼1000 participants per age group, of which 300 per age group would also donate biological samples (blood and urine samples were collected only from children aged 18 mo and 4 y). A sample size of 300 blood samples per age group was estimated to be adequate to assess prevalence of vitamin D status (<30 nmol/L) with a precision of ±3.5% (80% power and α = 0.05). Approximately, 300 stool samples per age group (from 1000 children) was estimated to be enough to identify a 50% increase of pathogen prevalence associated with a risk factor (80% power and α = 0.05). Only children living in the vicinity of 1 of the country’s 7 Environmental and Occupational Medicine (EOM) clinics, where blood sampling would be performed, were asked to donate blood and urine samples. Because we expected that too few participants would live close enough to an EOM clinic, the population living within 50 km of an EOM clinic was oversampled by 15%. The oversampling was proportional to the number of children aged 0–4 y living in each EOM region (Figure 1; [13]). For children aged 4 y, 7800 children who turned 4 between August 2021 and May 2022 were drawn. Because participation rates for 4-y-old children turned out to be lower than expected, the sample size for children aged 18 mo was increased to 9000 children. This sample included children turning 18 mo between September 2022 and May 2023. For children aged 9 mo, a sample of 5500 children turning 9 mo between September 2023 and May 2024 was drawn.

#### Recruitment

The recruitment process was similar for all age groups. Participants were invited by letter. In the letter, individual codes for each legal guardian were included to be able to consent or decline via a web-based form. Guardians could choose to participate in 1 or more parts of the survey and confirmed their choice and identity with digital identification, BankID. In 2022, 99% of those nationally registered in Sweden aged 18–65 y had access to BankID [14]. There was also an option to provide consent using the codes included in the letter and 2% consented without digital identification. Guardians who did not respond to the invitation and for which telephone numbers had been identified (75% of the children in the sample), were reminded by telephone call or text message up to 4 times. The remaining 25% without a known telephone number were sent a reminder invitation letter.

#### Incentives

Depending on which parts of the survey participants chose, they were offered gift cards ranging from 200 SEK (∼€17/$18: food diary and questionnaires only) to 600 SEK (∼€51/$54: all parts, including physical activity and blood samples). They were also offered simple personalized feedback on the food diary and physical activity measurements. Those who participated in blood sampling also received feedback on plasma ferritin concentrations. For children aged 9 mo, gift cards of 100 SEK (∼€8.5/$9) were given for questionnaires and additional 100 SEK for stool samples.

### Dietary assessment method

#### RiksmatenFlex

The RiksmatenFlex method was developed for the previous Swedish national dietary survey and has been validated in adolescents [15]. The web-based method is flexible and assesses diet by either 24-h recall or a food diary (RiksmatenFlexDiet) as well as questionnaires (RiksmatenFlexQ). The website is responsive and can therefore be accessed by smartphone, computer, or tablet. Participants provide their e-mail and mobile phone number, which are used to send automatic reminders to complete questionnaires and recalls/food diaries.

#### RiksmatenFlexDiet

EFSA recommends that a food diary is used to assess food intake in young children [3], and thus, this method was chosen for children aged 18 mo and 4 y. The food diary was filled in on 2 nonconsecutive days. The participants were instructed to start the diary on a weekend day and the second day was randomly assigned to a weekday 4–11 days later. If the child attended daycare, guardians were instructed to ask daycare staff to fill out a paper diary, which was then transferred to the web-based food diary by the guardians.

In the food diary, guardians registered the time, place (e.g., at home and preschool) and meal type (e.g., breakfast and snack). They then searched for foods or dishes and added portion sizes based on photographs or household measures. Automatic reminders were prompted when no drinks were added to a meal or when the meal consisted of <4 food items. At the end of the food diary, a list of often forgotten foods such as bread and butter, sweets, and vitamin D supplement were added.

In RiksmatenFlexDiet, a suitable food list containing a selection of food items from the Swedish Food Agency’s food composition database is chosen for the target study population. For Riksmaten Young Children, some adjustments were made in the food database and in the web-based method. The food composition database was updated with foods often consumed by toddlers and young children, based on statistics of sales in this food segment as well as gathering menus from preschools. Culture-specific foods of 3 major immigration groups (Syria, Iraq, and Somalia) were also added (M. Lentjes, Örebro University, personal communication, 2023). The nutrient content of foods that needed to be added or updated were then either chemically analyzed, borrowed from other food composition databases, or calculated from recipes. Food portions were also adjusted in order to accommodate smaller portion sizes (e.g., teaspoons) and other consistencies (e.g., purées). The user-friendliness was tested among a small convenience sample of guardians of children aged 1 to 5 y and revised where necessary. The final food list in Riksmaten Young Children comprised 1218 foods and composite dishes. Of these, 43% were chemically analyzed foods and 57% were calculated food items based on analyzed foods. The diary is linked to the food composition database, which enables automatic assessment of nutrient intakes.

#### RiksmatenFlexQ

The questionnaires were self-administered and could be completed during a 4-week period from the start of the survey. They were divided into 4 chapters with ∼45 questions in each chapter. Each chapter took ∼10 min to complete, and participants did not have to complete all questions in 1 sitting. The questionnaires covered topics such as the child’s birth country, information about guardians, family structure (number of children and adults in the household), height and weight of the child, information on food allergies, physical activity, food frequency questions, breast feeding, food choices, use of different food packaging and contact materials, intakes of rarely consumed food, drinking water, and use of dietary supplements and antibiotics. For children aged 9 mo, similar questionnaires were used, with the addition of an extended food frequency questionnaire in order to capture food intake.

### Physical activity

Physical activity was measured with tri-axial accelerometers (ActiGraph GT3X) among children aged 18 mo and 4 y. The accelerometer was worn around the waist for 7 consecutive days, while awake (except for when taking baths or other activities in water). A paper form was filled in with the time of day when the child got up in the morning and went to bed at night for each measurement day. Participants also answered questions about physical activity in the questionnaire.

### Biomonitoring

Blood and urine samples were collected in a subsample of children aged 18 mo and 4 y at clinics run by regional divisions of EOM. Sweden’s 7 EOMs are spread across Sweden (Lund, Gothenburg, Linköping, Örebro, Stockholm, Uppsala, and Umeå), providing a nationally distributed sample geographically. All children in the study sample living in a municipality at most 50 km from an EOM were invited to provide blood and urine samples (*n* = 10,051; 18 mo: *n* = 5506; 4 y: *n* = 4544]). The EOM in Uppsala could not participate, so blood and urine samples of children living in Uppsala were therefore collected by a nurse employed by the Swedish Food Agency. The participants were invited to the clinic for sampling when they had completed the first day of the 2-d food diary. Nonfasting venous blood was collected from the back of the hand or the bend of the arm by registered nurses or other certified personnel. The site was anesthetized using a patch with lidocain and prilocain. Up to 12.5 mL blood was collected (6 mL for serum, 4.5 mL for plasma, 2 mL whole blood), centrifuged (2200*g* for 10 min) after waiting ≥30 min (and maximum 5 h), pipetted into aliquots and stored at −80 °C at the EOM. The whole blood sample was pipetted to aliquots without being centrifuged. The samples were then transported to the Swedish Food Agency, where they were stored at −80 °C until analysis. The samples are used to derive information about status of both nutrients (e.g., ferritin, folate, and vitamin D analogs in plasma and sodium and iodine in urine), and toxic substances (e.g., heavy metals in whole blood, perfluoroalkyl and polyfluoroalkyl substances and mycotoxins in serum, as well as mycotoxins and phenolic compounds in urine). Plasma ferritin was analyzed continuously during the study to be able to offer the participants feedback on their iron status. The participants were asked to collect a urine sample in a 50-mL tube at home the same morning as the EOM clinic visit and to bring the urine sample to the clinic where it was aliquoted and stored at −80 °C until transportation to Swedish Food Agency. Urine was not collected from children who wore diapers.

Stool samples were collected at home in all age groups. When ∼300 samples had been collected from children aged 4 y, participants were no longer invited to provide samples. For children aged 18 mo, approximately, for every second, participant was asked to donate a stool sample until ∼300 samples was reached. For children aged 9 mo, the goal was also to collect 300 stool samples. Collection tubes were sent to the guardians, who collected samples from the children and sent them back to the Swedish Food Agency by post the same day or the day after. Two stool samples were collected from each participant: 1 in a container without any additives (Feces container; Sarstedt) for molecular analyses and 1 container with Cary-Blair medium (FecalSwab; Copan) for bacterial culturing. Upon arrival at the Swedish Food Agency, the samples were stored in a refrigerator until taken care of, most often the same, or the following, day. Glycerol (∼10%) was added to the FecalSwab containers, vortexed, and split into 2 subsamples by removing 1 mL of the supernatant to a cryo tube (microtube 2 mL; Sarstedt). All subsamples were stored at −20 °C before analyses. At the time of the stool sampling, a short paper questionnaire about gastrointestinal disease within the last 28 d was completed and returned with the samples.

### Anthropometry

Weight and height of the children were collected in the questionnaire. Guardians were asked to provide the weight and height measured at the last visit to a Children’s Healthcare Centre along with the date of the visit. In Sweden, children visit a Children’s Healthcare Centre on a regular basis from birth to age 5 y. Three of the visits take place at ages 8–10 mo, 18 mo, and 4 y [16]—in other words, close to the time of the data assessments in Riksmaten Young Children. If the child had not been to a scheduled visit recently, guardians were asked if they had measured or could estimate weight and height at home. It was noted whether the data were measured, and by whom, or estimated. Those that did not have data from a visit were asked whether it was okay to contact them again later to check if they had new data. Weight status was assessed according to International Obesity Task Force (IOTF) [17] to allow for comparisons with national statistics, as this is the reference used by the Swedish child healthcare system [16].

### Dropout analysis

Participating families were compared with national statistics in corresponding target age groups. Information on sex, birth country of child and parents, highest attained education and annual income in the household, family structure and number of children aged <6 y, as well as type of municipality was provided by Statistics Sweden at the group level.

## Results

This section describes results for the age groups 4 y and 18 mo.

### Participation

**FIGURE 2:**
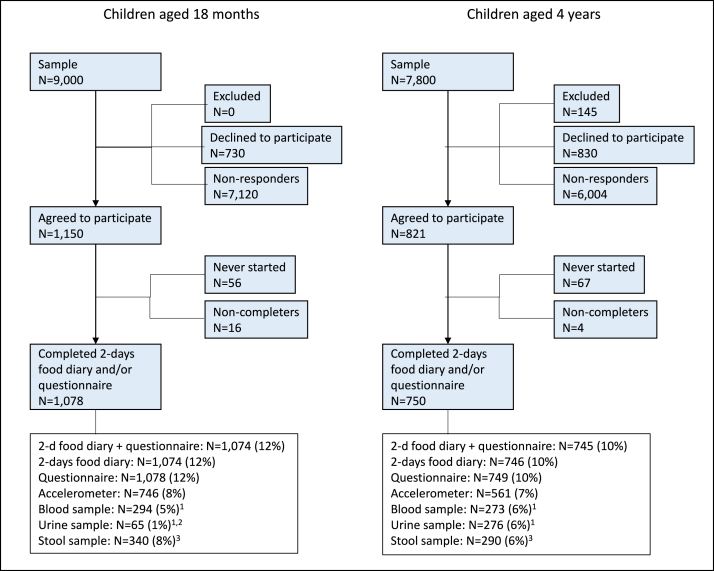
Study flow chart of Riksmaten Young Children. ^1^Collected in a subsample (18 mo: *n* = 5506; 4 y: *n* = 4544). ^2^Urine was only sampled in children aged 18 mo who were not using diapers. ^3^Collected in a subsample (18 mo: *n* = 4257; 4 y: *n* = 4608).

Figure 2 gives an overview of the study of Riksmaten Young Children in the form of a flow chart. In total, 16,800 children aged 18 mo or 4 y were invited and 1828 (11%) completed the questionnaire and/or 2-d food diary. The final participation rate was 12% (*n* = 1078) in the 18-mo-old and 10% (*n* = 750) in the 4-y-olds groups. A very high proportion of participants (99%) completed the food diary and/or questionnaire once starting data collection (Figure 2). Nine participants were excluded because their food diaries did not fulfill the following criteria: *1*) 2 registration days, *2*) reported energy intake of >100 kJ per day if the day was reported as a normal day. Of the registered days, 27% included food consumption in preschool [18 mo: *n* = 538 d (25%); 4 y: *n* = 454 (31%)].

The willingness to participate in the optional components was relatively high. Accelerometer data were assessed in 71% of the eligible participants (*n* = 1307) (Figure 2). Stool samples were collected from 60% of eligible participants (*n* = 630), and blood and/or urine samples were assessed among 51% of eligible participants (*n* = 593) (Figure 2).

The participants were distributed all over Sweden and in proportions reflecting the distribution of all Swedish children aged 0–4 y between the EOM regions (Figure 1). The children donating blood and urine were also well distributed over the EOM regions, but the participation rate was slightly lower in Linköping and higher in Stockholm (Figure 1).

### Population characteristics

The mean age of the children was close to the target ages. The mean age of the 18-mo-old group was 1.5 ± 0.1 y (minimum–maximum: 1.4–1.9 y); the mean age of the 4-y-old group was 4.1± 0.1 y (minimum–maximum: 3.9–4.6 y). Girls made up 48% and boys 52%, in agreement with the corresponding Swedish population (Table 1). Usable data on weight and height recorded at the most recent health care center visit were available for 90% (*n* = 1641) of the children; 2% (*n* = 42) measured weight and height at home. Weight status according to IOTF [17] was calculated for children who provided weight and height measured between ages 3.5 and 4.5 y (*n* = 629): 13.5% with underweight (*n* = 85), 77.6% with normal weight (*n* = 488), 7.3% with overweight (*n* = 46), and 1.6% with obesity (*n* = 10). IOTF does not have cutoffs for weight status of children aged <2 y, so weight status was not calculated for children aged 18 mo. The majority of the questionnaires were completed by the mother (80%), and the mean age of the participating guardians was 35.5 y.

**TABLE 1:** Socioeconomic characteristics of participants in Riksmaten Young Children Dietary Survey.

	18 mo				4 y			
	Participants (*n* = 1078)		Swedish population (*n* = 88,831)		Participants (*n* = 750)		Swedish population (*n* = 126,478)	
	*n*	%	*n*	%	*n*	%	*N*	%
Child’s sex								
Girls	512	47	43,270	49	366	49	61,453	49
Boys	566	53	45,561	51	384	51	65,025	51
Child’s birth country								
Sweden	1075	100	88,077	99	736	98	121,100	96
Outside Sweden	3	0	754	1	14	2	5378	4
Parents’ birth country								
Both or one parent born in Sweden	848	79	55,972	63	594	79	76,663	61
One parent born in Sweden, 1 outside Sweden	153	14	12,555	14	107	14	16,148	13
Both or only parents born outside Sweden	77	7	20,304	23	49	7	33,667	27
Highest education attained at household level								
Primary school/unknown (≤9 y)	38	4	12,082	14	5	1	8949	7
High school (10–12 y)	289	27	35,060	39	97	13	38,937	31
University degree or higher (>12 y)	751	70	41,689	47	648	86	78,592	62
Annual household income (SEK)								
<550,000	215	20	30,471	34	132	18	44,551	35
550,000 to <800,000	401	37	31,950	36	265	35	43,536	34
≥800,000	462	43	26,410	30	353	47	38,391	30
Family structure								
Parents not living together	26	2	4978	6	36	5	13,795	11
Parents living together	1014	94	80,025	90	691	92	106,799	84
Single parent	38	4	3828	4	23	3	5884	5
No. of children <6 y in the household								
1	601	56	48,224	54	386	51	70,202	56
2	453	42	36,224	41	336	45	49,375	39
≥3	24	2	3861	4	28	4	6454	5
Type of municipality								
Large cities and municipalities near large cities	392	36	36,134	41	283	38	49,455	39
Medium-sized towns and municipalities near medium-sized towns	499	46	35,042	39	346	46	48,393	38
Smaller towns/urban areas and rural municipalities	187	17	17,655	20	121	16	28,630	23

Data are based on register data provided by Statistics Sweden. Highest household education: primary school/unknown, both parents have primary school or unknown education; high school; at least 1 parent has a high school degree; university degree, at least 1 parent has a university degree. Household income is defined as the sum of incomes before taxes. Number of children is the number of children registered on the same address as the participating child and includes the participating child. Unknown number of children in the Swedish population are excluded (18 mo: *n* = 522; 4 y: *n* = 447). Classification of Swedish municipalities by Swedish Association of Local Authorities and Regions was based on child’s registered address.

Response rates were lower in families where the child or both guardians were born outside of Sweden, in families where the highest household education was less than university degree level, and in families with a total disposable yearly income of <550,000 SEK (Table 1). All geographic regions and types of municipalities were represented (Table 1, Figure 1). Participation rates were lower among children from single-guardian households and where guardians were not living together.

### Recruitment

**FIGURE 3:**
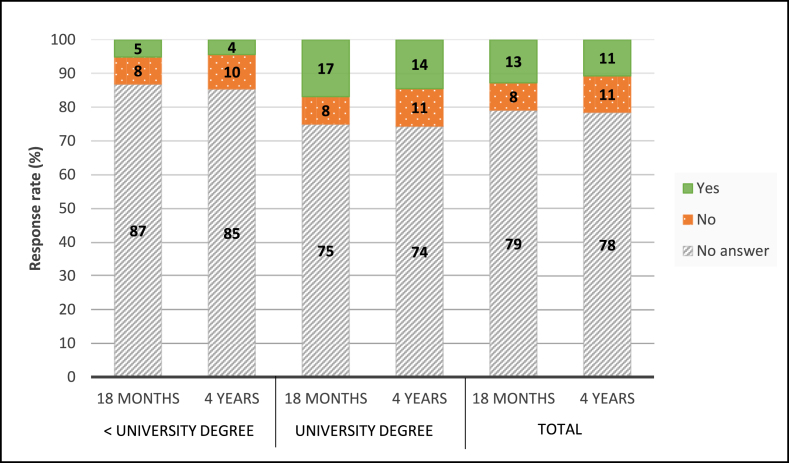
Responders and nonresponders in Riksmaten Young Children divided by age group and household education level. University degree, at least 1 parent has a university degree. Unknown education is included in less than university degree.

**FIGURE 4:**
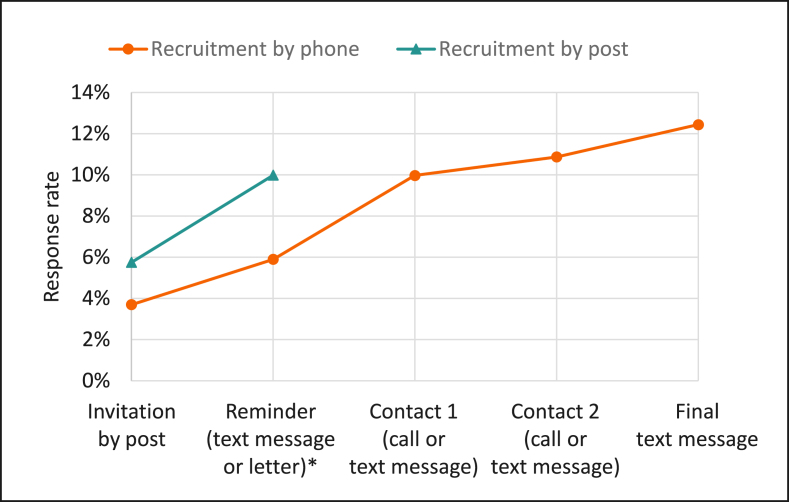
Contact attempts needed for consent of participants (*n* = 1971) in Riksmaten Young Children divided by recruitment via post and telephone or post only. Phone numbers were available for 75% (*n* = 12,568) of the sample. Sample without phone numbers (25%, *n* = 4087) were sent a reminder letter following the initial invitation letter. ∗Reminder was sent by phone for the sample with phone numbers and by post for the sample without telephone numbers.

The majority of the invited families did not respond (79%) (Figure 2), that is, they neither accepted nor declined participation despite repeated contacts by telephone or post. The nonresponse rate was higher among households with education less than university degree level compared than that among households with a university degree (Figure 3). A telephone number for at least 1 guardian was available for 75% of the sample (*n* = 12,568). These families were therefore recruited by telephone following the invitation letter by post. We were able to identify slightly fewer telephone numbers for the sample without a university degree (73%) than those with a university degree (77%). The sample without telephone numbers (25%, *n* = 4087) was sent a reminder letter instead. Hence, they were not contacted the same number of times. Figure 4 shows the cumulative participation rate among participants giving consent to the study according to whether the recruitment was done by post and telephone or by post only. The participation rate was 12% for recruitment via post and telephone and 10% via post only. Only 35% of all participants giving consent responded after the first invitation and repeated contacts were needed for most participants. The participation rate increased after each contact attempt. For participants with a telephone number, the highest increase was seen after the first call attempt, although the call was not answered in most cases and was therefore immediately followed by a text message (Figure 4). There were no differences between household education levels regarding at which recruitment stage the participants consented.

## Discussion

Riksmaten Young children is a national dietary survey in infants, toddlers, and children, collecting detailed information on food consumption and nutrient intake using food diaries and questionnaires. In subgroups, objectively measured physical activity and data on biomarkers analyzed in blood, urine, and stool samples were also collected. Hence, Riksmaten Young Children provides a broad picture of food intake, exposure, and nutrient status of young children using several different assessment methods, and can be used for improving healthy food habits, assessing food safety, and guiding policymakers. To the best of our knowledge, few previous national studies in toddlers and young children have been performed [8,18], especially including all abovementioned measurements. Most nationally representative biomonitoring studies are conducted in children aged ≥3 y [[19], [20], [21], [22], [23]]. Although there are studies that also include newborns [24], in general, there are limited data in children aged <2 y [25] and a lack of information on nutrient deficiency in toddlers [6].

Although the predefined sample size goal of 1000 was reached in 18-mo-old children, and of around 300 in both age groups for blood and stool samples, the overall participation rate (11%) was lower than expected. In the previous national survey in children, participation rates were 64% among 4-y-old children [9]. However, that survey was conducted in 2003, and a lower response rate, 20%, has been seen in a more recent Norwegian national dietary survey in 4-y-old children from 2016 [26]. Large variations of response rates (23%–99%) have been reported in other European dietary surveys [18]. Decreasing tendency to participate in studies is a well-known problem in Sweden as well as elsewhere [[27], [28], [29]], and difficulty to recruit participants is the main issue in many studies. Younger adults (i.e., the target population of guardians in Riksmaten Young Children) are especially likely to respond at low rates [27,28]. This may have contributed to the lower response rate in Riksmaten Young Children than that in other studies conducted by Swedish authorities [28,29]. Another reason could be that dietary registration is more burdensome compared with the questionnaires used in those other studies. Because dietary intake is the primary aim of Riksmaten Young Children, this part could not be voluntary. Disappointingly, we were not able to increase the response rate despite several contact methods, number of attempts, and several incentives. The participants were offered gift cards, individual feedback (on diet, physical activity, and plasma ferritin concentrations), as well as the flexibility to choose which study parts to conduct.

The data collection of 4-y-old children was initiated during the first year of the COVID-19 pandemic, which may well have affected the participation rates, in ways that are difficult to predict. In Sweden, preschools did not close during the pandemic, but many guardians worked from home, and more time was spent at home with children because preschools did not allow children with even slight symptoms to attend. Socializing with older relatives was not recommended, which may have increased the burden on guardians. On the contrary, more time spent at home may have enabled participation.

Because nonresponse is often systematic, low-participation rates suggest that results may be skewed in some way. In our case, it is likely with respect to socioeconomic position. The dropout analysis showed that the participants (and their guardians) were more often born in Sweden, had more guardians with a university degree, and higher disposable income than the Swedish population. This is a well-known problem in surveys, and similar skewness has been reported in national dietary surveys in Denmark [30] and Norway [26]. Results where socioeconomic position could be strongly associated with the outcome of interest must be interpreted considering this bias. However, the study population was representative regarding the Swedish population considering sex, type of municipality, geographic distribution over Sweden, and number of children in the household. The study population also included children of all weight categories, including 9% with overweight or obesity (compared with 13% in the latest compilation of anthropometric data from the child health care visits in Sweden) [31]. In line with that data, a higher prevalence of overweight and obesity in girls than that in boys was observed.

Several steps were taken before and during the study to reduce the risk of bias and/or to increase the quality. A random sample was drawn from all children of aged 9 mo, 18 mo, and 4 y using national registries, meaning that all children had the same possibility to be invited to the study. The recruitment was standardized, and a customer relationship management system was used for a quality-assured process. Two nonconsecutive prospective 1-d food diaries were used as dietary assessment method, as recommended in this study population by EFSA [3]. The recommended follow-up interview [3] was replaced by an automatic control using questions on frequently forgotten foods such as ketchup, water, and candy. The EFSA guidance does, however, recommend ≥7 d between the 2 nonconsecutive data collection days. In Riksmaten Young Children, the time interval was between 4 and 11 d. The reason is based on experience from the pilot study of the previous national dietary survey Riksmaten adolescents 2016–2017, where the participants were more likely to drop out if a longer interval between the collection days were applied. The web-based method RiksmatenFlex is validated in adolescents [15] regarding energy expenditure (accelerometer-estimated), wholegrain food consumption (plasma alkylresorcinol), and consumption of fruit and vegetables (plasma carotenoids), and a validation study in young children aged 2–5 y is ongoing. Food diary data were also complemented with questionnaires to gather information such as demography, seldomly consumed food items, and dietary behavior.

Despite the low-participation rate, the partial nonresponse (when respondents provide incomplete information) was low, and 99% of the participants completed the study once starting the data collection. Only 20 (1%) of all participants logging in to RiksmatenFlex did not complete the 2 d of food records or the questionnaire (Figure 2). This indicates that the tool RiksmatenFlex is user-friendly despite the fact that a food diary is a burdensome assessment. It also indicates motivated participants, especially because the guardians needed to involve the preschools in the assessment.

The proportion of invited guardians actively saying no did not differ by household education, but households without a university degree were less likely to respond to the invitation at all. We identified slightly less telephone numbers for participants without a university degree. These could therefore not be contacted by telephone and instead reminders were sent by post. This could partly have contributed to a lower participation rate among those with lower socioeconomic position, although this study group is difficult to recruit irrespectively of recruitment strategy [26,[32], [33], [34]]. Interestingly, similar response rates (10%) were found for participants only reminded by a letter and those reminded with a text message and 1 contact attempt via telephone. One could therefore speculate that sending a reminder letter could be a less burdensome and more time-effective recruitment intervention. However, the response rate after the first invitation letter was higher in the sample without than that in those with telephone numbers (6% and 4%, respectively). Hence, this conclusion must be interpreted with caution. Furthermore, the participation rate increased following additional recruitment interventions in the sample with telephone numbers. Overall, 65% of all participants giving consent needed repeated contacts before they chose to participate. Repeated contacts more than a first reminder did not specifically increase inclusion of participants with lower education level and did therefore not reduce the skewness.

The strengths of this study include the national perspective capturing the dietary habits in young children all over Sweden using a user-friendly assessment method containing a food list and portion sizes adapted for the target population. Another is the use of several methods (food diary, questionnaire, accelerometer, and biomonitoring) giving detailed information about dietary habits, food intake, exposure, and nutrient status. The biomonitoring part is unique in its inclusion of a national representative sample of young children and of particular interest because biomonitoring of toxic compounds has received increased attention as an important tool to support risk assessment, and chemical policy and regulation [35,36]. Further, because we have assessed food consumption both at home and in preschools, we have the possibility to investigate the impact of preschools on food quality and socioeconomic discrepancies in food habits. We have previously shown that school meals can help mitigate diet-related social inequalities in dietary intake in adolescents [7]. The main limitation of the study is risk of biased results regarding factors related to socioeconomic position (i.e., the respondents were more educated than the general Swedish population). Another drawback is that RiksmatenFlex is only available in Swedish, which excluded guardians not fluent in Swedish.

Riksmaten Young Children provides a substantial amount of national-level information in toddlers and young children, a population where food habits are established and with higher risk of adverse effects from exposure to toxic substances and enteric viruses. The data are representative with respect to sex, geography, and family structure and will be used in a variety of areas such as improving healthy food habits, assessing food safety, and guiding policymakers. The underrepresentation of households with lower socioeconomic position must be considered when generalizing results associated with these factors to the Swedish population.

## Author contributions

The authors’ responsibilities were as follows – LM, HB, EP, JPS, SL, AKL, JO, EWL: designed research; LM, HB, EP, JPS, SL, JO, KS, EL: conducted research; HB, LM, EP: analyzed data; HB, LM, EP: wrote the paper; LM: had primary responsibility for final content; and all authors: have read and approved the final manuscript.

## Funding

The biomonitoring in the survey (sampling of blood and urine) was partly financed by the Swedish Environmental Protection Agency.

## Data availability

Data described in the manuscript will be made available when the data have been anonymized, planned for 2029.
